# Molecular signature of renal cell carcinoma by means of a multiplatform metabolomics analysis

**DOI:** 10.1016/j.bbrep.2022.101318

**Published:** 2022-08-04

**Authors:** Marta Kordalewska, Renata Wawrzyniak, Julia Jacyna, Joanna Godzień, Ángeles López Gonzálves, Joanna Raczak-Gutknecht, Marcin Markuszewski, Piotr Gutknecht, Marcin Matuszewski, Janusz Siebert, Coral Barbas, Michał J. Markuszewski

**Affiliations:** a Department of Biopharmaceutics and Pharmacodynamics, Medical University of Gdańsk, Al. Gen. Hallera 107, 80-416, Gdańsk, Poland; b Metabolomics Laboratory, Clinical Research Centre, Medical University of Białystok, ul. Jana Kilińskiego 1, 15-089, Białystok, Poland; c CEMBIO, Centre of Metabolomics and Bioanalysis, San Pablo CEU University, Madrid, 28003, Spain; d Department of Urology, Medical University of Gdańsk, ul. Mariana Smoluchowskiego 17, 80-214, Gdańsk, Poland; e Department of Family Medicine, Medical University of Gdańsk, Dębinki 2, 80-211, Gdańsk, Poland

**Keywords:** Untargeted metabolomics, Renal cell carcinoma, Multiplatform approach, Complementary analytical techniques, Mass spectrometry

## Abstract

Renal cell carcinoma (RCC) is a disease with no specific diagnostic method or treatment. Thus, the evaluation of novel diagnostic tools or treatment possibilities is essential. In this study, a multiplatform untargeted metabolomics analysis of urine was applied to search for a metabolic pattern specific for RCC, which could enable comprehensive assessment of its biochemical background. Thirty patients with diagnosed RCC and 29 healthy volunteers were involved in the first stage of the study. Initially, the utility of the application of the selected approach was checked for RCC with no differentiation for cancer subtypes. In the second stage, this approach was used to study clear cell renal cell carcinoma (ccRCC) in 38 ccRCC patients and 38 healthy volunteers. Three complementary analytical platforms were used: reversed-phase liquid chromatography coupled with time-of-flight mass spectrometry (RP-HPLC-TOF/MS), capillary electrophoresis coupled with time-of-flight mass spectrometry (CE-TOF/MS), and gas chromatography triple quadrupole mass spectrometry (GC-QqQ/MS). As a result of urine sample analyses, two panels of metabolites specific for RCC and ccRCC were selected. Disruptions in amino acid, lipid, purine, and pyrimidine metabolism, the TCA cycle and energetic processes were observed. The most interesting differences were observed for modified nucleosides. This is the first time that the levels of these compounds were found to be changed in RCC and ccRCC patients, providing a framework for further studies. Moreover, the application of the CE-MS technique enabled the determination of statistically significant changes in symmetric dimethylarginine (SDMA) in RCC.

## Highlights

• Multiplatform untargeted metabolomics analysis was applied for selection of tentative diagnostic indicators of RCC.
• LC-MS, GC-MS and CE-MS techniques were utilized for analysis of urine samples collected from RCC and ccRCC patients.
• Alterations in amino acid, purine, and pyrimidine metabolism, as well as TCA cycle and energy processes, were observed.

## 1 Introduction

Renal cell carcinoma (RCC) is diagnosed in over 400,000 patients annually and has one of the highest mortality rates among urinary tract cancers [1]. The likelihood of disease development is two times higher in men than in women [2]. Epidemiological (smoking, obesity, comorbidities, unhealthy diet, some medications) exposure to carcinogens or genetic factors is considered to be the main risk factor for RCC [3]. The four main syndromes von Hippel–Lindau disease (VHL, caused by pathogenic variants in VHL); hereditary leiomyomatosis and renal cell cancer (HLRCC, caused by pathogenic variants in FH); Birt-Hogg-Dubé syndrome (BHD, caused by pathogenic variants in FLCN); and hereditary papillary renal carcinoma (HPRC, caused by pathogenic variants in MET) are considered to be responsible for hereditary RCC subtypes [4]. Unfortunately, there is no noninvasive treatment available for RCC, and only surgical intervention produces successful results. Moreover, common symptoms of RCC development and progression are nonspecific and include hematuria, flank pain or masses that can be palpably detected through abdominal covers [5,6]. Therefore, this disease is commonly diagnosed during diagnosis of other abdominal diseases with the use of specialized and expansive diagnostic tools such as ultrasonography (USG), computed tomography (CT), or magnetic resonance imaging (MRI) [7]. Due to these issues, RCC and its common subtypes, such as clear cell renal cell carcinoma (ccRCC), papillary renal cell carcinoma (pRCC), or chromophobe renal cell carcinoma (chRCC) [8], require further studies on their biochemical background or potential diagnostic and treatment methods.

The metabolome constitutes a complex set of compounds, namely, metabolites, characterized by a wide range of physicochemical properties [9]. Determination of such a diverse group of compounds is still a great challenge for today's science. It is well known that there is no single analytical technique that can cover the whole set of metabolites present in biological materials [10]. Therefore, the application of complementary analytical platforms allows the detection of many compounds, even those with extremely different physicochemical characteristics, that are often derived from the same biochemical pathway.

While the application of liquid chromatography with reversed-phase separation allows for the analysis of compounds of a wide polarity range, highly polar metabolites cannot be separated through this approach [11]. Thus, the application of hydrophilic interaction liquid chromatography or capillary electrophoresis is recommended [11,12]. For the analysis of compounds that can be volatilized after derivatization over a wide polarity range, the gas chromatography technique is suitable [13].

In this study, a multiplatform approach was applied to evaluate metabolite alterations associated with RCC and one of its most common subtypes, ccRCC. Liquid and gas chromatography, as well as capillary electrophoresis coupled with mass spectrometry, have been used for the determination of metabolites present in urine samples collected from RCC patients and healthy volunteers.

To date, many studies have been performed in the field of RCC and its subtypes. Metabolomics approaches, both untargeted [[14], [15], [16], [17], [18], [19], [20]] and targeted [[21], [22], [23], [24], [25]], with LC–MS [17,18,26,27], GC–MS [20,28,29] and NMR [15,16,30,31] have been applied. In six studies, at least two techniques were combined to increase metabolome coverage [14,19,22,24,[32], [33], [34]]. In the majority of cases, LC/MS and GC/MS measurements were performed [22,24,32,34]. An interesting approach was implemented by Ganti and colleagues [22], where GC/MS analysis was supported with LC/MS measurement using HILIC and RP chromatography. Similarly, Okegawa and colleagues [33] used GC/MS with ion pairing LC/MS and LC/MS-based lipidomics. However, to the best of the authors’ knowledge, this is the first study on RCC in which three MS-based platforms were applied with the use of LC, GC and CE to discover unique metabolic alterations.

## 2 Materials and methods

### 2.1 Sample collection

Urine samples were collected from the Department of Urology (RCC patients) and the Department of Family Medicine (healthy controls) at the Medical University of Gdańsk in Poland. The characteristics of the patients enrolled in the study are presented in the Supplementary Materials Tables 1 and 2. The project was performed following the principles embodied in the Declaration of Helsinki and executed according to the Ethical Committee of the Medical University of Gdańsk (number of consent: NKBBN/8/2016). Urine samples were collected from RCC (Fuhrman grade from 1 to 4) and ccRCC (Fuhrman grade 2 and 3) patients before surgical intervention and any treatment related to the disease. The classification of patients into groups was based on the histopathology results. For participants selection following criteria were applied: age, sex, body mass index (BMI), no smoking status and exclusion of comorbidities. However, during biochemical interpretation any changes related to hypertension, diabetes, metabolic syndrome, smoking or diet were taken into consideration. The group of healthy volunteers did not receive medication at the time of sample collection: each patient was declared healthy. After collection, urine samples were immediately frozen and stored at −80 °C. Directly before analytical measurements, urine samples were thawed at room temperature.

### 2.2 Urine sample preparation

#### 2.2.1 HPLC-TOF/MS

Prior to LC–MS analyses, thawed urine samples were vortexed for 1 min and then centrifuged at 2469×*g* for 15 min at 4 °C. Next, 500 μl of supernatant was diluted in 500 μl of deionized water. Subsequently, the samples were centrifuged at 2469×*g* for 15 min at 4 °C. The supernatants were filtered directly into dark glass vials with nylon filters with a pore size of 0.22 μm. Afterward, samples were analyzed with the use of the RP-HPLC-TOF/MS technique.

#### 2.2.2 GC-QqQ/MS

After thawing, urine samples were vortexed for 1 min and then centrifuged at 2469×*g* for 15 min at 4 °C. A volume of 200 μl of supernatant was transferred to a glass tube and treated with urease solution in water (30 units/1 ml). Reduction of excess urea was enhanced by incubation at 37 °C for 30 min. Afterward, 800 μl of cold methanol (kept for 30 min at −80 °C) and 10 μl of a solution of pentadecanoic acid in methanol (1 mg/ml) were added. Samples were vortexed for 5 min and then centrifuged at 2469×*g* for 15 min at 4 °C. Two hundred microliters of supernatant was transferred to glass inserts in dark glass vials and evaporated to dryness at 30 °C for 2 h. Subsequently, 30 μl of methoxyamine in pyridine (15 mg/ml) was added. Next, the samples were vortexed for 10 min and incubated for 16 h at room temperature in the dark. Afterward, 30 μl of BSTFA with 1% TMCS was added to each sample, and 5 min of vortex mixing was performed. The samples were incubated for 1 h at 70 °C. As the last step, 70 μl of hexane or heptane was added, and samples were vortex-mixed for 10 min.

#### 2.2.3 CE-TOF/MS

The sample pretreatment procedure included the addition of 100 μl of 20.0 mM formic acid with 0.4 mM methionine sulfate to 100 μl of thawed urine sample. Next, the samples were vortexed for 1 min and centrifuged for 20 min at 4 °C at 16,000×*g*. A total of 100 μl of the obtained supernatant was transferred to a vial and analyzed.

### 2.3 Quality control sample (QC) preparation

Quality control samples (QCs) were used to control the reproducibility of sample preparation procedures, analytical methods and system stability during the sequence run. QCs were prepared as a mixture of equal volumes (50 μl) of each analyzed urine sample. For all analytical techniques, QCs were prepared with the same procedure as that used for real urine samples and analyzed at the beginning and repeatedly during a sequence run.

### 2.4 Untargeted metabolic fingerprinting

Analyses with the use of the CE-TOF/MS technique were performed in the Centre of Metabolomics and Bioanalysis (CEMBIO) at CEU San Pablo University in Madrid, while HPLC-TOF/MS and GC-QqQ/MS analyses were carried out in the Department of Biopharmaceutics and Pharmacodynamics at the Medical University of Gdańsk.

#### 2.4.1 HPLC-TOF/MS

Untargeted metabolomics analysis of urine samples was performed with a 1200 HPLC coupled with a 6224 TOF/MS system (Agilent Technologies, Germany) equipped with a dual electrospray ionization source (Dual-ESI). A Zorbax Extend-C18 Rapid Resolution HT column (2.1 × 100 mm; 1.8 μm) was used. The injection volume was 2 μl, and the mobile phase flow rate was 0.35 ml/min. The time of analysis was 18 min, with 10 min column equilibration. RP-HPLC separation was performed in gradient elution mode using mobile phases: A – 0.1% formic acid in water and B – 0.1% formic acid in acetonitrile. The gradient elution program was as follows: 0–6 min from 98% to 80% A, 6–9 min from 80% to 55% A, 9–14 min from 55% to 2% A and from 14 to 18 min 2% A. The column temperature was set to 35 °C. The drying gas temperature and flow rate were 350 °C and 11 L/min, respectively, with the nebulizer pressure set to 50 psi. The capillary, fragmentor and skimmer voltages were 3250 V, 150 V and 65 V, respectively. Data were collected in scan mode with *m/z* ranging from 50 to 1200 in both positive and negative ionization modes. Additionally, the analyses were performed using the in-source fragmentation method with the fragmentor voltage set to 120 V, 160 V and 200 V, with other parameters unchanged.

#### 2.4.2 GC–QqQ/MS

Analytical measurements of prepared urine samples, with the use of gas chromatography coupled with triple quadrupole mass spectrometry, were performed with the GC TQ8030 system (Shimadzu, Japan) equipped with an electron ionization (EI) ion source. Compound separation was performed with a Zebron ZB-5MS column (30 m × 0.25 mm, 0.25 μm; Phenomenex, USA) with helium as a carrier gas. A volume of 1 μl of each sample was injected into the instrument with the injector temperature set to 250 °C. The temperature gradient program for the first set of samples was 40 °C (5 min), 40 °C–261.5 °C (3 °C/min), 261.5 °C (1 min), 261.5 °C–320 °C (15 °C/min), and 320 °C (10 min). The total time of analysis was 93.73 min. For the second set of samples (diluted in heptane), the temperature gradient program was 60 °C (1 min), 60 °C–320 °C (8 °C/min), and 320 °C (5 min), with a total analysis time of 38.5 min. The carrier gas flow rate and pressure were set to 10 ml/min and 53.5 kPa, respectively. The transfer line temperature was 300 °C. Mass spectra were collected in scan mode with *m/z* ranging from 50 to 600. The ion source temperature was set to 200 °C. At the beginning of each sequence run, the mixture of alkanes (from C10 to C40, with even carbon numbers) was analyzed and then applied for data processing (retention index (RI) calculation, retention time (RT) alignment and metabolite identification).

#### 2.4.3 CE-TOF/MS

Analyses of previously prepared urine samples, with the use of capillary electrophoresis coupled with time-of-flight mass spectrometry, were performed with a 7100 CE coupled with 6224 TOF/MS system (Agilent Technologies, Germany) equipped with an ESI ion source with a sheath liquid delivery system. Separation of the compounds was performed with a fused silica capillary (50 μm × 100 cm; Agilent Technologies, USA). Before each analysis, the capillary was flushed with BGE (0.8 ml/L, formic acid in 10% methanol in water) for 5 min at 950 mbar. Prepared urine samples were injected into the capillary for 50 s under 50 mbar pressure. Next, BGE was injected for 10 s under 100 mbar pressure. Compound separation was performed under a pressure of 25 mbar, voltage of 30 kV and amperage of 22 μA. The sheath liquid was delivered to the ion source with a flow rate of 0.6 ml/min. The drying gas temperature and flow rate were 200 °C and 10 L/min, respectively, with the nebulizer pressure set to 10 psi. The capillary, fragmentor and skimmer voltages were 3500 V, 125 V and 65 V, respectively. The mass spectrometer was operated in scanning mode with a m*/z* range from 50 to 1000.

### 2.5 Data processing and statistical analyses

The LC–MS data pretreatment procedure included extraction of all detected signals with the use of the Molecular Feature Extraction (MFE) algorithm in MassHunter Qualitative Analysis software (B.04.00) and DA Reprocessor (B.04.00) (Agilent Technologies). The feature extraction was performed based on the charge, isotopic pattern, presence of dimers, and adducts (+H, +Na, +K in positive ionization mode; –H, +HCOO in negative ionization mode; and neutral water loss –H_2_O). The feature alignment was performed using Mass Profiler Professional software (B.02.01) (MPP; Agilent Technologies). The 1% shift in the retention time and 20 ppm error in mass detection were considered acceptable. The created data matrix was filtered based on the quality assurance criteria, including feature presence in 50% of QC samples, coefficient of variance (CV) in QCs lower than 20%, and presence in 80% of the studied groups (RCC or ccRCC patients *vs.* healthy controls). Feature intensities were normalized using the MS group useful signal (MSGUS) method [35].

Data obtained with the GC–MS technique were prepared with the use of the Automated Mass Spectral Deconvolution and Identification System (AMDIS; National Institute of Standards and Technology, USA). The data pretreatment protocol included RI calculation, RT alignment (applied RT window: ± 0.1 min), and compound identification based on the NIST11 spectra library. Variable filtration was performed using Mass Profiler Professional B.02.01 (MPP; Agilent Technologies) with the application of quality assurance criteria (presence in 50% of QC samples, CV for QCs <30%, presence in 80% in one of the studied groups, namely, RCC or ccRCC patients *vs.* healthy controls). Data normalization was performed using the MSGUS method.

CE-MS data were reprocessed using MassHunter Profinder (B.06.00) software (Agilent Technologies). Data extraction with recursion was performed. During MFE algorithm application, the proton addition (+H) and neutral water loss were included. The data alignment was performed with a 1% shift in retention time and a 20 ppm error in mass acceptance. Visual inspection of the mass spectra and extracted ion chromatograms allowed the elimination of false-positive results in the created data matrix and correction of erroneous or missed peak integrations. This strategy minimized missing values generated during data processing; thus, the features were filtered in the MPP due to CV in QC samples lower than 20% and presence in 80% in one of the studied groups. Feature intensities were normalized with the use of the MSGUS method.

Univariate statistical analysis was performed with the MATLAB 2013b environment (Mathworks, USA). After data normality and unity of variance were checked with the Shapiro–Wilk and Levene's tests, respectively, the Student's *t*-test, Welch's test, or Mann–Whitney U test was applied. Features differentiating the studied groups (RCC or ccRCC patients *vs.* healthy controls) were selected based on an adjusted *p* value lower than 0.05 after correction for multiple comparisons (Benjamini–Hochberg FDR – False Discovery Rate).

Multivariate statistical analysis was performed with SIMCA-P 13.03 and 15.0.1 software (Umetrics, Sweden). Principal component analysis (PCA) was performed to check the stability of the systems. The presence of outliers was checked with the T-square Hotelling test with a 95% confidence interval. Next, orthogonal partial least squares discriminant analysis (OPLS-DA) was performed. The variables discriminating the studied groups were selected based on the variable importance into projection (VIP) value and selectivity ratio (SR) value equal to or higher than 1.

Three values describing feature significance were used: corrected *p* value, VIP, and SR. Features meeting one of these three conditions were considered to be differentiating between the compared groups.

### 2.6 Metabolite annotation

LC–MS and CE-MS detected features were annotated using the in-source fragmentation approach. For this, additional analyses were performed, keeping all LC and MS parameters but enhancing the fragmentor voltage to intentionally induce fragmentation of metabolites in the ion source. For LC–MS analyses, the fragmentor voltage was set to 120 V, 160 V, and 200 V, while for CE-MS analyses, it was set to 175 V [36]. Putative annotation of metabolites detected with the use of the LC–MS and CE-MS techniques was performed with the use of freely available databases and tools for their overview, such as the Human Metabolome Database (HMDB; http://www.hmdb.ca/), METLIN (https://metlin.scripps.edu/), KEGG (https://www.genome.jp/kegg/), and LIPIDMAPS (https://www.lipidmaps.org/) through CEU Mass Mediator 3.0 (http://ceumass.eps.uspceu.es/) [37]. Annotation was based on the molecular mass, retention time, isotopic pattern, and in-source fragmentation pattern. In the case of the GC–MS technique, identification of compounds was performed by comparison of the obtained MS spectra with the NIST11 spectra library. Interpretation of the roles of the selected metabolites in RCC and ccRCC was performed based on the available literature data, HMDB, and KEGG databases.

## 3 Results

Urine untargeted metabolomics with the use of three complementary analytical techniques, HPLC-TOF/MS, GC-QqQ/MS, and CE-TOF/MS, was performed. The project was divided into two experiments. In the first one, urine samples were collected from patients diagnosed with RCC and healthy controls and analyzed with the use of LC–MS, GC–MS, and CE-MS techniques. In the second one, patients with ccRCC and healthy volunteers were included, and urine samples were analyzed using LC–MS and GC–MS techniques. This approach allowed us to propose two panels of metabolites representing characteristic changes for the studied disease types or subtypes.

### 3.1 Urine metabolic fingerprinting

#### 3.1.1 HPLC-TOF/MS

Urine untargeted metabolomics analyses using HPLC-TOF/MS in scan mode after data pretreatment, including extraction, alignment, and filtration steps, resulted in the creation of a data matrix that was subjected to statistical calculations. For the first experiment, the data matrix included 573 and 194 features for positive and negative ionization modes, respectively. Similarly, for the second experiment, the datasets included 205 and 145 features.

The stability of the apparatus during the sequence run was verified by building PCA models. The system stability and method reproducibility were confirmed by clustering QC samples with respect to the obtained models (Supplementary Material Fig. 1).

**Fig. 1:**
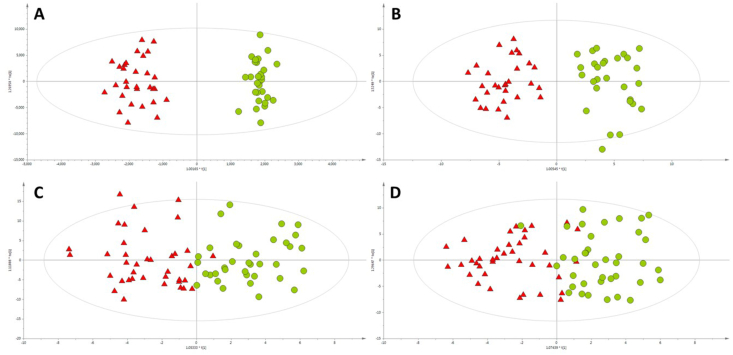
OPLS-DA models built for LC-TOF/MS data collected for A – first ESI + experiment (R^2^ = 0.963, Q^2^ = 0.535); B – first ESI- experiment (R^2^ = 0.882, Q^2^ = 0.614); C – second ESI + experiment (R^2^ = 0.703, Q^2^ = 0.474); D – second ESI- experiment (R^2^ = 0.656, Q^2^ = 0.398). Green circles and red triangles represent healthy volunteers and RCC/ccRCC patients, respectively. (For interpretation of the references to color in this figure legend, the reader is referred to the Web version of this article.)

The discrimination of the studied samples into the group of RCC/ccRCC patients or healthy individuals was studied based on OPLS-DA models, presented in Fig. 1.

The results of compound selection with the putatively annotated metabolites are presented in the Supplementary Materials (Tables 3, 4, 5and 6).

#### 3.1.2 GC-QqQ/MS

Urine untargeted metabolomics analyses using GC-QqQ/MS in scan mode after data pretreatment, including deconvolution, alignment, and filtration steps, resulted in the creation of a data matrix that was subjected to statistical analyses. The data matrix included 72 and 63 features for the first and second experiments, respectively.

The system stability and method reproducibility were confirmed by clustering QC samples on PCA models (Supplementary materials Fig. 2).

**Fig. 2:**
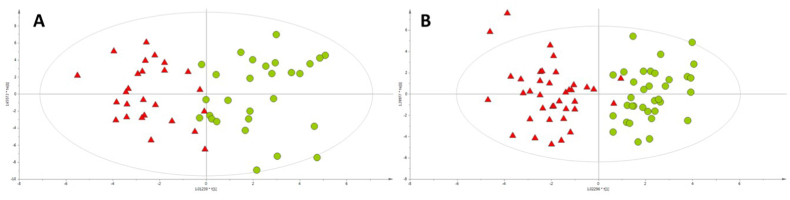
OPLS-DA models built for GC-QqQ/MS data collected for A – first experiment (R^2^ = 0.713, Q^2^ = 0.347) and B – second experiment (R^2^ = 0.780, Q^2^ = 0.545). Green circles and red triangles represent healthy volunteers and RCC/ccRCC patients, respectively. (For interpretation of the references to color in this figure legend, the reader is referred to the Web version of this article.)

The discrimination of the studied samples with respect to the group of RCC/ccRCC patients or healthy individuals was studied based on OPLS-DA models, as presented in Fig. 2.

The results of compound selection with the putatively annotated metabolites are presented in the Supplementary Materials (Tables 7 and 8).

#### 3.1.3 CE-TOF/MS

Urine untargeted metabolomics analyses using CE-TOF/MS in scan mode after data pretreatment, including extraction, alignment, and filtration steps, resulted in the creation of a data matrix including 310 features, which were then subjected to statistical analysis.

The system stability and method reproducibility were also confirmed by clustering QC samples with respect to the obtained models (Supplementary materials Fig. 3).

**Fig. 3:**
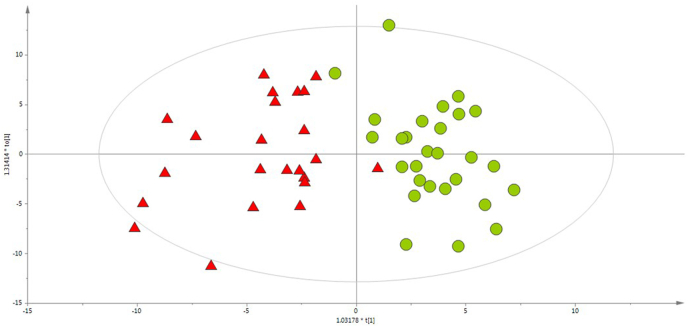
OPLS-DA models built for CE-ESI(+)-TOF/MS data (R^2^ = 0.738, Q^2^ = 0.537). Green circles and red triangles represent healthy volunteers and RCC patients, respectively. (For interpretation of the references to color in this figure legend, the reader is referred to the Web version of this article.)

The discrimination of the studied samples into the group of RCC patients or healthy individuals was studied based on OPLS-DA models, presented in Fig. 3.

The results of compound selection with the putatively annotated metabolites are presented in the Supplementary materials (Table 9).

The application of the CE-MS technique was useful for distinguishing between symmetric and asymmetric forms of dimethylarginine (SDMA, ADMA). The relative migration time values for SDMA and ADMA were calculated by dividing their migration time values in each urine sample by the migration time values of the corresponding internal standard (methionine sulfone). The calculated values were verified with a library of standards built into the CEMBIO laboratory and provided tentative identification.

### 3.2 Biochemical interpretation

Putative annotation of metabolites provided information about metabolic changes related to both RCC and ccRCC. The created list of compounds was then used for the interpretation of observed disturbances in the context of disease development molecular pathogenesis.

In the first experiment (RCC patients *vs.* healthy controls), changes mainly in amino acid, purine, pyrimidine, fatty acid metabolism, the Krebs cycle and energy production were observed. Moreover, some changes in metabolite composition were suspected to be related to gut microbiota metabolism. The list of metabolites with corresponding biochemical pathways is presented in Table 1.

**Table 1:** Statistically significant metabolites selected by comparison of samples collected from RCC patients and healthy controls with the biochemical pathways from which they originate and the analytical techniques used for their detection.

Metabolic pathway	Regulation	Metabolites
**Phenylalanine metabolism**	↓	hippuric acid (LC+, LC-), phenylalanine (GC), phenylacetylglutamine (LC+)
**Tryptophan metabolism**	↑	tryptophan (LC-)
	↓	tryptophan (GC), indolelactic acid (LC-), dihydroxyquinoline (LC+), hydroxytryptophan (GC), picolinic acid (GC)
**Tyrosine metabolism**	↑	succinylacetone (LC-)
	↓	tyrosine (LC+)
**Histidine metabolism**	↑	ribosylhistidine (CE+), formiminoglutamic acid (CE+), formylisoglutamine (CE+)
	↓	histidine (CE+), methylhistidine (CE+), hydantoinpropionic acid (CE+)
**Lysine metabolism**	↓	lysine (GC)
**Glycine, serine, and threonine metabolism**	↓	glycine (CE+), aminopropanol (CE+), sarcosine (CE+), guanidineacetic acid (CE+), creatine (CE+), threonine (GC), diaminopropane (GC)
**Arginine and proline metabolism**	↑	creatinine (CE+), symmetric dimethylarginine (CE+)
	↓	creatinine (GC), acetylarginine (GC), argininic acid (CE+)
**Alanine, aspartic acid, and glutamic acid metabolism**	↓	alanine (CE+), glutamic acid (GC), aspartic acid (GC)
**Pentose phosphate cycle**	↓	gluconic acid (GC)
**Pentose and glucuronic acid metabolism**	↓	pentose (GC)
**Purine metabolism**	↑	uric acid (LC-), dimethylguanosine (LC+, LC-, CE+)
	↓	adenosine (GC), methylguanosine (GC), deoxyguanosine (GC), glutamine (GC)
**Pyrimidine metabolism**	↑	uridine (LC-), pseudouridine (LC-), dihydrouridine (CE+), cytidine (CE+), deoxyuridine (CE+), acetylcytidine (LC+)
	↓	deoxycytidine (GC)
**Ascorbic acid metabolism**	↓	glucaric acid (GC)
**Krebs cycle**	↑	aconitic acid (CE+)
	↓	citric acid (LC+, LC-, GC), isocitric acid (LC+, LC-), pyruvic acid (LC-), succinic acid (GC), acetamidobutanoic acid (CE+)
**Pterin metabolism**	↑	biopterin (CE+)
**Glycerophospholipid metabolism**	↓	acetylcholine (GC)
**Sphingolipid metabolism**	↑	sphinganine (LC+),
	↓	sphingosine (GC)
**Fatty acid metabolism**	↑	hydroxysebacic acid (LC-), acetylcarnitine (LC+), hexanoylcarnitine (LC+), octanoylcarnitine (LC+), methylglutarylcarnitine (LC+),
	↓	methylsuberic acid (LC-), capryloylglycine (LC+), acylcarnitine (LC+), ethylmalonic acid (GC), nonadecanoic acid (GC), nicotinuric acid (GC), propionylcarnitine (CE+), aminocaprylic acid (CE+), aminooxohexanoic acid (CE+)
**Cholesterol biosynthesis**	↓	mevalonic acid (GC)
**Steroid hormones biosynthesis**	↑	cortolone glucuronide (LC-)
	↓	deoxycortisol (GC)
**Collagen metabolism**	↑	galactosylhydroxylysine (CE+)
**Butyric acid metabolism**	↓	hydroxyglutaric acid (GC)
**Gut microbiota metabolism**	↓	trimethylamine oxide (CE+), hydroxyhippuric acid (LC-), acetylglycine (GC), formylglycine (GC)

Table legend: ↓ - downregulation RCC *vs.* healthy controls, ↑ - upregulation RCC *vs.* healthy controls.

In the second experiment, wherein urine metabolic profiles of ccRCC patients were compared with profiles of urine samples collected from healthy volunteers, primarily the changes in amino acid metabolism, purine metabolism, and energy flux were observed. The list of metabolites with corresponding biochemical pathways is presented in Table 2.

**Table 2:** Statistically significant metabolites selected in the comparison of ccRCC patients and healthy controls, characterized by the biochemical pathways from which they originated and the analytical techniques used for detection.

Metabolic pathway	Regulation	Metabolites
**Phenylalanine metabolism**	↑	phenylacetylglutamine (LC+, LC-)
	↓	hippuric acid (LC+, LC-, GC)
**Tryptophan metabolism**	↓	indolelactic acid (LC-)
**Ascorbic acid metabolism**	↑	threonic acid (GC)
**Purine metabolism**	↑	methyladenosine (LC+), uric acid (LC-)
**Pentose and glucuronic acid metabolism**	↑	pentose (GC), arabitol (GC)
**Amino sugar and nucleotide sugar metabolism**	↑	acetylglucosamine (GC)
**Galactose metabolism**	↑	galactinol (GC)

Table legend: ↓ - downregulated ccRCC *vs.* healthy controls, ↑ - upregulated ccRCC *vs.* healthy controls.

## 4 Discussion

It is well known that untargeted metabolomics is a useful tool for the evaluation of differences in metabolic profiles resulting from pathological processes when compared to the healthy status. In this study, untargeted metabolomics was applied to detect metabolic changes in RCC and ccRCC in comparison with healthy subjects. The application of three complementary analytical techniques allowed us to propose a large panel of metabolites with different physicochemical properties. The potential roles of these compounds in molecular pathomechanisms were confirmed or explained based on the available literature data.

RCC can be caused by mutations in the VHL, MET (MET Proto-Oncogene, Receptor Tyrosine Kinase), FLCN (Folliculin), fumarate hydratase, succinate dehydrogenase, TSC1 (Tuberous sclerosis 1), TSC2 (Tuberous sclerosis 2), and TFE3 (Transcription Factor Binding To IGHM Enhancer 3) genes, some of which are involved in cellular respiration and energy metabolism [38]. As an example, VHL gene mutation results in overproduction of HIF-1 (hypoxia-inducible factor 1), leading to the Warburg effect and other changes in metabolic processes in cancer cells [39]. In ccRCC, changes in energy metabolism regulated by HIF-1 are observed due to the loss of function of the VHL gene. The excessive uptake of glucose by cancer cells, intensified glycolysis and lactic acid production, and reduced impact of oxidative phosphorylation were reported by Semenza et al. [40]. Fumarate hydratase, which is deactivated in papillary renal cell carcinoma, is responsible for the hydration of fumaric acid to malic acid, which is a part of the Krebs cycle. This enzyme inactivation results in reactive oxygen species (ROS) production and HIF-1 stabilization with the use of glucose. As a result, oxidative glycolysis intensifies, and the importance of mitochondrial respiration is reduced [41]. Another process observed in ccRCC progression is the inactivation of histone H3 lysine trimethyltransferase (SETD2). This leads to elevated levels of Krebs cycle acids, namely, aspartic, malic, succinic, fumaric, and α-ketoglutaric acid, in cancer tissue. The loss of activity of SETD2 was also suggested to be linked with pyruvic acid metabolism. Deactivation of pyruvate dehydrogenase is frequently observed in cancer cells. Differences in the concentrations of metabolites of the Krebs cycle can be a result of pyruvate to acetyl coenzyme A (acyl-CoA) conversion disorder. Additionally, disturbances in mitochondrial metabolism, as well as glucose and fatty acid metabolism, can occur [42]. The metabolite changes caused by RCC or ccRCC in this study were mostly related to amino acid, purine, pyrimidine, fatty acid, and carbohydrate metabolism, as well as the Krebs cycle and energy metabolism.

Cancer cells are characterized by very fast division resulting in uncontrolled proliferation. Due to this fact, energy and essential building blocks must be constantly provided. The accumulation of free amino acids and changes in their metabolism can be observed when compared to healthy tissue [43]. The changes in amino acid metabolism observed in the study are consistent with the data reported by Hakimi et al. [44]. For instance, a reduced level of tryptophan in urine samples collected from RCC patients can be a result of its intensified catabolism. Two enzymes are mainly responsible for this process: indoleamine 2,3-dioxygenase and tryptophan dioxygenase. The shift of tryptophan metabolism to the production of immunosuppressive metabolites, mentioned by Platten et al. [45], is beneficial for cancer development. Liu et al. [17] highlighted the role of tryptophan metabolism in RCC progression. The differences in amino acid metabolism were also found to be dependent on the RCC subtype by Jing et al. [46].

SDMA is a product of the methylation of arginine residues and is considered to be a better marker of kidney function than creatinine [47]. This molecule is excreted mainly by the kidneys and shows a significant relationship with glomerular filtration rate (GFR) [48]. SDMA was also found to have potential for the diagnosis of chronic kidney disease (CKD) [49]. The correlation with interleukin (IL-6) and tumor necrosis factor α (TNF-α), indicating the proinflammatory function of this compound in CKD, was reported by Schepers et al. [50]. The role of SDMA as a risk factor for cardiovascular disease was also highlighted [51]. In our study, an elevated level of SDMA was found in the urine of RCC patients, which may be interesting in the context of renal function disruption due to the presence of a tumor. Moreover, it should be emphasized that the application of the CE-MS method enabled us to separate SDMA from ADMA in a simple manner, which is quite difficult and requires a derivatization process when the HPLC–MS technique is utilized [52].

Biopterin biosynthesis is strictly related to the metabolism of phenylalanine, tyrosine, and tryptophan. The elevated level of this metabolite can be a result of incorrect activity of tyrosine or tryptophan hydroxylase, disturbed levels of neurotransmitters, and/or enzymatic hyperactivity. The use of phenylalanine resources results in the production of tetrahydrobiopterin, and ultimately biopterin [53]. Moreover, changes in hippuric acid metabolism are related to phenylalanine metabolism. However, it should be mentioned that this metabolite can also be of dietary, microbial or environmental origin [54]. In our study, a decreased level of hippuric acid in urine samples was observed.

The Warburg effect, described above, is also characterized by an excessive supply of glucose when compared to healthy tissues. Glucose is fermented to lactic acid, even in the presence of oxygen. Excess carbohydrate constitutes a carbon source utilized for the synthesis of nucleotides, lipids, and peptides, which are necessary for cancer growth and proliferation [55]. The glycolysis process is strictly related to the pentose-phosphate pathway, as well as serine synthesis and metabolism [56]. Disturbances in the Krebs cycle, energetic processes, and the metabolism of different classes of lipids and fatty acids are consistent with assumptions of the Warburg effect.

In the reported study, changes in the composition of fatty acids and lipids were observed in the urine of RCC patients compared to the healthy group. The intensification of fatty acid synthesis is a response to an increased supply of cancer cells with demands for substances used as energy resources, cell membrane components, and signaling compounds [57]. Overexpression of FAS is related to the development of cancer, as this enzyme is involved in fatty acid biosynthesis [58]. Overexpression of FAS was found to positively correlate with the stage of RCC and to decrease patients’ chances for recovery [59]. There is also a link between the Krebs cycle and fatty acid synthesis in cancer cells. Citric acid, after transportation from mitochondria to the cytosol by transport proteins, is transformed by ATP-citrate lyase to oxaloacetic acid and acyl-CoA used in lipid synthesis [60]. Fatty acids are substrates for sphingolipid synthesis. This group of compounds can both support (sphingosine-1-phosphate) and inhibit (ceramides) the carcinogenesis process [61,62]. In the case of kidney cancer with VHL gene mutation, the overproduction of sphingosine-1-phosphate was reported, as this metabolite can induce proliferation, cell movement, and angiogenesis [63]. This fact can explain the reduced sphingosine signal observed in the reported study. As observed in this study, a higher concentration of sphinganine may be a result of decreased ceramide production.

Although the accumulation of free cholesterol is toxic, an 8-fold higher concentration of total cholesterol and a 35-fold higher concentration of its ester form can be observed in ccRCC cells than in healthy kidney tissue [64]. Cancer cells have developed a protective mechanism associated with hyperactivity of acetyl-CoA cholesterol acetyltransferase, an enzyme responsible for the catalysis of the production of nontoxic cholesterol ester from free cholesterol and fatty acetyl-CoA [65]. Mevalonic acid is one of the compounds used for cholesterol production. In this study, an elevated level of this metabolite was detected in the urine of ccRCC patients. Mevalonic acid can be produced from 3-hydroxy-3-methylglutaric acid by 3-hydroxy-3-methylglutaryl coenzyme A reductase (HMG-CoA), which regulates cholesterol synthesis [66].

Acylcarnitines are compounds that mediate fatty acid β-oxidation. According to Huang et al. [67], HIF-1 inhibits the fatty acid β-oxidation process by reducing medium chain acyl-CoA dehydrogenase activity. In the study performed by Schmidt-Sommerfeld et al. [68], higher concentrations of hexanoylcarnitine and octanoylcarnitine were detected in comparison with other medium chain acylcarnitines. This study was based on the analysis of urine samples collected from patients with medium chain acyl-CoA dehydrogenase dysfunction. Higher levels of acylcarnitines and palmitoylcarnitine were also found in the urine of ccRCC patients, and mechanisms leading to this are possible reasons for the chemoresistance of this disease [22,46].

Threonic acid, an elevated level of which was observed in ccRCC included in this study, participates in ascorbate and aldarate metabolism. Intensified oxidative stress resulting in excessive degradation of ascorbic acid and protein glycation can lead to threonate overproduction [69,70]. Glucaric acid also participates in ascorbate and aldarate metabolism. Its decreased level was observed in our study in RCC patients. According to Walaszek et al. [71], reduced excretion of this metabolite in both human and rat models of RCC was observed. However, the biological role of this compound is still not clearly or fully explained.

Disturbances in the activity of l-hydroxyglutaric acid dehydrogenase are a reason for the accumulation of 2-hydroxyglutaric acid in cancer cells during ccRCC progression. In particular, accumulation of its L enantiomer is observed [72,73]. Cancers characterized by elevated levels of 2-hydroxyglutaric acid exhibit simultaneous decreases in the level of 5-hydroxymethylcytosine in genomic DNA [24]. The reduced level of 2-hydroxyglutaric acid in the urine of RCC patients in the reported study may be a consequence of the aforementioned dysregulations.

As a result of RNA metabolism and the process of DNA oxidative damage, nucleosides are created. Moreover, posttranscriptional changes in nucleic acids, namely, methylation, isomerization, dehydrogenation, acetylation, and hydroxylation, can lead to the creation of modified nucleosides. Nucleosides are released due to the activity of phosphatases and ribonucleases. Basic nucleosides (adenosine, guanosine, cytidine, uridine) are reutilized for RNA synthesis or metabolized to uric acid, β-alanine, and β-aminoisobutyric acid. However, modified nucleosides do not undergo this process and in the unchanged form are directly removed with urine. It was observed that the intensification of RNA degradation and metabolism takes place during the process of carcinogenesis. The consequences of this fact are elevated levels of urine nucleosides, both basic and modified. In 1985, Koshida et al. [74] found elevated levels of pseudouridine in kidney cancer tissue and in urine samples collected from cancer patients. Moreover, in 1991, Rasmuson et al. [75] confirmed the aforementioned observations. Additionally, these metabolites are potential biomarkers of oxidative stress [76]. The role of nucleosides was confirmed in the development of cancers of the urinary tract [77], breast [78], gallbladder [79] and liver [80]. In the reported study, elevated levels of uric acid and several nucleosides were observed. Nevertheless, there remains a need for an explanation of the detected decreased levels of methylguanosine, deoxyguanosine, and deoxycytidine. To the best of the authors’ knowledge, this is the first metabolomics study in which altered levels of nucleosides were observed in the context of RCC molecular pathogenesis.

Galactosylhydroxylysine is a product of collagen metabolism and is utilized as a marker of bone resorption [81]. Current reports describe that its concentration strictly correlates with breast cancer bone metastases [82]. In a reported study, an elevated level of galactosylhydroxylysine was observed in RCC patients in comparison with healthy controls.

The literature offers some evidence for a relationship between RCC and steroid hormone activity. It was previously reported that abnormal expression of receptors for these hormones can occur in RCC tissue [83,84]. However, there is still a lack of explanation for the potential role and reasons for the level differences (RCC *vs.* healthy controls) of cortolone glucuronide or deoxycortisol in RCC development.

In this study, some metabolites, in the form of products of bacterial metabolism, were found to be statistically significant. Decreased levels of formylglycine, trimethylamine oxide, hydroxyhippuric acid, and acetylglycine were observed in urine samples. Currently, the relationship between elevated levels of plasma trimethylamine oxide and chronic kidney disease is highlighted. However, there are some questions as to whether this compound accumulation is a reason for or a consequence of the disease [[85], [86], [87]]. An altered level of trimethylamine oxide was also observed in the prostate [88], stomach [89] and colon cancer [90]. Derosa et al. [91] highlighted the role of gut microbiota in RCC resistance to immunotherapy. However, further studies are required to evaluate the roles of specific bacteria and explain the underlying mechanisms.

Creatine, and ultimately creatinine, are created as a result of choline metabolism. Alterations in energy processes and choline metabolism in RCC result in altered levels of both compounds. Creatinine concentration in urine and plasma is used for calculation of clearance, which, from the clinical point of view, is used for the determination of kidney function [92]. In studies on diseases involving kidney failure, it is necessary to estimate the influence of mechanistic destruction (*i.e.,* caused by the tumor) on filtration. However, this influence should be distinguished from biochemical processes and related metabolite changes observed in the studied disorder. Data normalization should reduce both biological and analytical variability.

**Fig. 4:**
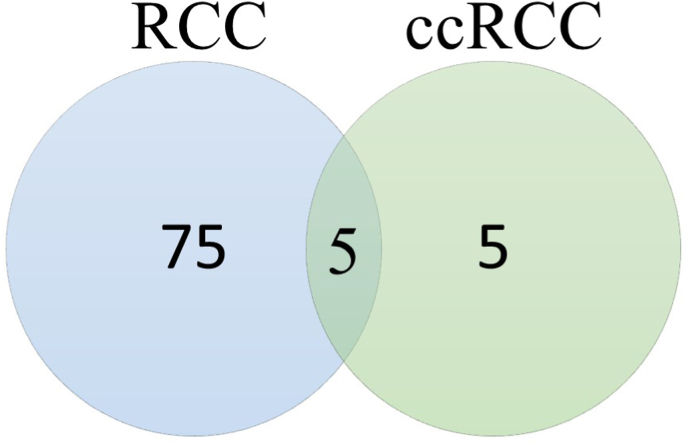
Venn-diagram presenting number of different and common metabolites between RCC and ccRCC groups.

**Fig. 5:**
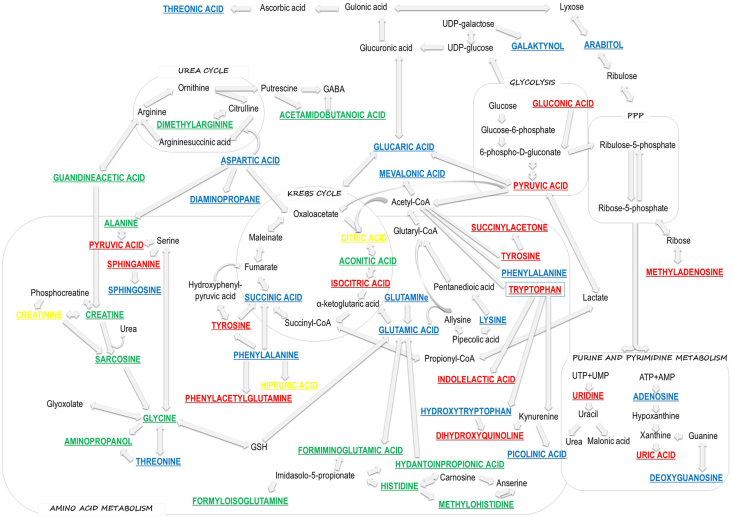
Changes in metabolic processes detected with the use of complementary analytical platforms (red color – metabolites detected with LC–MS, blue – GC–MS, green – CE-MS, yellow – with more than one technique). (For interpretation of the references to color in this figure legend, the reader is referred to the Web version of this article.)

The creation of such a large panel of metabolites found to be changed due to RCC or ccRCC presence was possible by application of complementary analytical techniques. Only three metabolites whose levels were significantly different in the studied groups were detected by the use of more than one analytical technique. Furthermore, only five metabolites (phenylacetylglutamine, hippuric acid, indolelactic acid, uric acid, pentose) were common for RCC and ccRCC groups (Fig. 4). The biochemical changes and pathways observed in this study are presented in Fig. 5.

The obtained preliminary results constitute a great basis for further analysis with a quantitative approach, namely, targeted metabolomics. In this step, the confirmation of identity and the relationship between the concentration of compounds and RCC subtypes at different stages will be provided. The differences in the obtained panels of metabolites for RCC and ccRCC suggested the necessity of comparing the metabolic content for different subtypes of RCC to determine their specific patterns. This work opens new research possibilities for the explanation and evaluation of biochemical pathomechanisms underlying RCC.

## 5 Conclusions

The results of this preliminary study provide comprehensive insight into the biochemical processes underlying RCC and its most common subtype, ccRCC. The application of three complementary analytical techniques conferred the possibility of creating a list of metabolites that were observed to exhibit changes in levels of abundance due to disease status. Alterations in amino acid, purine, and pyrimidine metabolism, as well as TCA cycle and energy processes, were observed. The most interesting changes seem to be disturbances in the excretion of modified nucleosides such as pseudouridine and methylguanosine. To the best of the authors’ knowledge, this is the first time that these changes have been observed in the context of RCC or ccRCC. Moreover, the performed study confirmed an elevated level of SDMA in urine samples of RCC patients, which was possibly due to the application of the CE-MS technique. The obtained results constitute a promising beginning for targeted, quantitative analysis in which confirmation of metabolite identification and validation of the results would be performed.

## Declaration of competing interest

The authors declare that there are no conflicts of interest.
