# Supplementary material for: Molecular signature of renal cell carcinoma by means of a multiplatform metabolomics analysis
Source: Biochem Biophys Rep. 2022 Aug 4;31:101318. doi: 10.1016/j.bbrep.2022.101318 (PMC9363947; doi:10.1016/j.bbrep.2022.101318)
Supplement: Multimedia component 1 [file mmc1.doc]

**Supplementary materials**

Table 1. Characteristics of RCC patients and healthy controls involved in the first experiment.

| **Group** | **Number of patients (male/female)** | **Age [years]** | **BMI** |
| --- | --- | --- | --- |
|  |  | **Average (standard deviation)** | |
| **Healthy controls** | 29 (12/17) | 56.9 (±10.9) | 28.2 (±3.5) |
| **RCC patients** | 30 (17/13) | 61.6 (±10.1) | 28.2 (±3.7) |
| **Sum** | | | |
|  | 59 (29/30)  (*p* value*=0.24) | 59.3 (±10.7)  (*p* value=0.09) | 28.2 (±3.6)  (*p* value=0.96) |

* All *p* values were calculated using t-test or chi-square test

Table 2. Characteristics of ccRCC patients and healthy controls involved in the second experiment.

| **Group** | **Number of patients (male/female)** | **Age [years]** | **BMI** |
| --- | --- | --- | --- |
|  |  | **Average (standard deviation)** | |
| **Healthy controls** | 38 (22/16) | 62.9 (±8.5) | 26.2 (±2.1) |
| **ccRCC patients** | 38 (22/16) | 64.2 (±11.3) | 27.2 (±3.8) |
| **Sum** | | | |
|  | 76 (44/32)  (*p* value*= 0.56) | 63.6 (±10.0)  (*p* value=0.99) | 26.7 (±3.1)  (*p* value=0.18) |

* All *p* values were calculated using t-test or chi-square test

Table 3. Putatively annotated metabolites detected with the use of LC-ESI(+)-TOF/MS technique (experiment 1 – RCC *vs* healthy controls).

| **Mass [Da]*** | **RT**  **[min]** | **Metabolite** | **Mass error [ppm]** | **Formula** | **Formula match**  **Factor‖** | **Statistical test value** | **Regulation**  **(RCC *vs* healthy patients)** |
| --- | --- | --- | --- | --- | --- | --- | --- |
| 161.0476 | 7.38 | Dihydroxyquinoline | 0 | C_9_H_7_NO_2_ | 98% | SR=3.9 | ↓ |
| 179.0584 | 7.37 | Hippuric acid | 0 | C_9_H_9_NO_3_ | 99% | SR=11.6; VIP=7.0;  p FDR=0.017 | ↓ |
| 181.0738 | 7.97 | Tyrosine | 0 | C_9_H_11_NO_3_ | 92% | SR=1.2; p FDR=0.034 | ↓ |
| 192.0273 | 1.23 | Citric acid/ Isocitric acid | 1 | C_6_H_8_O_7_ | 99% | VIP=1.2 | ↓ |
| 201.1366 | 11.67 | Capryloylglycine | 0 | C_10_H_19_NO_3_ | 93% | p FDR=0.037 | ↓ |
| 203.1162 | 1.07 | Acetylcarnitine | 2 | C_9_H_17_NO_4_ | 94% | VIP=3.8 | ↑ |
| 259.1782 | 8.84 | Hexanoylcarnitine | 0 | C_13_H_25_NO_4_ | 97% | p FDR=0.041 | ↑ |
| 264.1111 | 7.66 | Phenylacetylglutamine | 0 | C_13_H_16_N_2_O_4_ | 99% | VIP=4.9 | ↓ |
| 273.2668 | 12.48 | Sphinganine | 0 | C_16_H_35_NO_2_ | 99% | VIP=3.5 | ↑ |
| 285.0962 | 4.72 | Acetylcytidine | 0 | C_11_H_15_N_3_O_6_ | 90% | p FDR=0.025 | ↑ |
| 287.2097 | 11.17 | Octanoylcarnitine | 0 | C_15_H_29_NO_4_ | 90% | p FDR=0.005 | ↑ |
| 289.1526 | 4.26 | Methylglutarylcarnitine | 0 | C_13_H_23_NO_6_ | 99% | p FDR=0.014 | ↑ |
| 301.2256 | 11.38 | Acylcarnitine | 0 | C_16_H_31_NO_4_ | 98% | VIP=1.7 | ↓ |
| 311.1232 | 5.61 | Dimethylguanosine | 0 | C_12_H_17_N_5_O_5_ | 99% | p FDR=0.027 | ↑ |

*Monoisotopic neutral mass; ‖ Matching factor was calculated using Mass Hunter Qualitative algorithm for formula match

SR: Selectivity Ratio; FDR: False Discovery Rate; VIP: Variable Importance in Projection.

Table 4. Putatively annotated metabolites detected with the use of LC-ESI(-)-TOF/MS technique (experiment 1 – RCC *vs* healthy controls)

| **Monoisotopic mass [Da]** | **RT [min]** | **Metabolite** | **Mass error [ppm]** | **Formula** | **Formula match**  **factor** | **Statistical test value** | **Regulation**  **(RCC *vs* healthy controls)** |
| --- | --- | --- | --- | --- | --- | --- | --- |
| 88.0166 | 1.09 | Pyruvic acid | 6 | C_3_H_4_O_3_ | 90% | p FDR=0.005 | ↓ |
| 158.0579 | 5.83 | Succinylacetone | 0 | C_7_H_10_O_4_ | 90% | SR= 1.1;  p FDR= 0.0002 | ↑ |
| 168.0290 | 1.00 | Uric acid | 4 | C_5_H_4_N_4_O_3_ | 94% | VIP=3.5;  p FDR=0.022 | ↑ |
| 179.0591 | 6.97 | Hippuric acid | 4 | C_9_H_9_NO_3_ | 95% | VIP=5.8,  p FDR=0.004 | ↓ |
| 188.1049 | 10.38 | Methylsuberic acid | 0 | C_9_H_16_O_4_ | 85% | p FDR=0.012 | ↓ |
| 192.0280 | 1.09 | Citric acid/ Isocitric acid | 5 | C_6_H_8_O_7_ | 95% | VIP=1.4 | ↓ |
| 195.0533 | 4.86 | Hydroxyhippuric acid | 1 | C_9_H_9_NO_4_ | 98% | p FDR=0.023 | ↓ |
| 204.0900 | 5.46 | Tryptophan | 0 | C_11_H_12_N_2_O_2_ | 85% | p FDR=0.023 | ↑ |
| 205.0739 | 10.16 | Indolelactic acid | 0 | C_11_H_11_NO_3_ | 97% | p FDR=0.024 | ↓ |
| 218.1155 | 9.01 | Hydroxysebacic acid | 0 | C_10_H_18_O_5_ | 98% | p FDR=0.008 | ↑ |
| 244.0699 | 0.94 | Uridine/Pseudouridine | 1 | C_9_H_12_N_2_O_6_ | 85% | p FDR=0.005 | ↑ |
| 311.1228 | 5.31 | Dimethylguanosine | 0 | C_12_H_17_N_5_O_5_ | 85% | p FDR=0.004 | ↑ |
| 542.2721 | 10.78 | Cortolone glucuronide | 1 | C_27_H_42_O_11_ | 92% | p FDR=0.002 | ↑ |

FDR: False Discovery Rate; VIP: Variable Importance in Projection

Table 5. Putatively annotated metabolites detected with the use of LC-ESI(+)-TOF/MS technique (experiment 2 – ccRCC *vs* healthy controls).

| **Monoisotopic mass [Da]** | **RT [min]** | **Metabolite** | **Mass error [ppm]** | **Formula** | **Formula match**  **factor** | **Statistical test value** | **Regulation**  **(ccRCC *vs* healthy controls)** |
| --- | --- | --- | --- | --- | --- | --- | --- |
| 179.0586 | 6.91 | Hippuric acid | 1 | C_9_H_9_NO_3_ | 99% | SR=1.6; VIP=1.6 | ↓ |
| 264.1113 | 7.23 | Phenylacetylglutamine | 0 | C_13_H_16_N_2_O_4_ | 90% | VIP=1.2 | ↑ |
| 281.1127 | 0.93 | Methyladenosine | 0 | C_11_H_15_N_5_O_4_ | 85% | p FDR=0.0001 | ↑ |

SR: Selectivity Ratio; FDR: False Discovery Rate; VIP: Variable Importance in Projection.

Table 6. Putatively annotated metabolites detected with the use of LC-ESI(-)-TOF/MS technique (experiment 2 – ccRCC *vs* healthy controls)

| **Monoisotopic mass [Da]** | **RT [min]** | **Metabolite** | **Mass error [ppm]** | **Formula** | **Formula match**  **factor** | **Statistical test value** | **Regulation**  **(ccRCC *vs* healthy controls)** |
| --- | --- | --- | --- | --- | --- | --- | --- |
| 168.0296 | 0.94 | Uric acid | 7 | C_5_H_4_N_4_O_3_ | 90% | VIP=2.3 | ↑ |
| 179.0598 | 6.70 | Hippuric acid | 9 | C_9_H_9_NO_3_ | 85% | SR=34.6; VIP=7.4;  p FDR= 0.00002 | ↓ |
| 205.0748 | 10.01 | Indolelactic acid | 4 | C_11_H_11_NO_3_ | 80% | p FDR=0.008 | ↓ |
| 264.1121 | 7.06 | Phenylacetylglutamine | 4 | C_13_H_16_N_2_O_4_ | 92% | VIP=2.3 | ↑ |

SR: Selectivity Ratio; FDR: False Discovery Rate; VIP: Variable Importance in Projection.

Table 7. Putatively annotated metabolites detected with the use of GC-EI-QqQ/MS technique (experiment 1 – RCC *vs* healthy controls).

| **Monoisotopic mass [Da]** | **RT**  **[min]** | **Metabolite** | **Formula** | **Statistical test value** | **Regulation (RCC *vs* healthy patients)** |
| --- | --- | --- | --- | --- | --- |
| 74.0843 | 30.57 | Diaminopropane | C_3_H_10_N_2_ | SR=1.5;  p FDR=0.009 | ↓ |
| 103.0269 | 23.94 | Formylglycine | C_3_H_5_NO_3_ | p FDR=0.011 | ↓ |
| 113.0589 | 29.61 | Creatinine | C_4_H_7_N_3_O | p FDR=0.037 | ↓ |
| 117.0426 | 34.28 | Acetylglycine | C_4_H_7_NO_3_ | SR=1.1 | ↓ |
| 118.0266 | 21.33 | Succinic acid | C_4_H_6_O_4_ | p FDR=0.012 | ↓ |
| 119.0582 | 33.33 | Threonine | C_4_H_9_NO_3_ | p FDR=0.010 | ↓ |
| 123.0320 | 32.86 | Picolinic acid | C_6_H_5_NO_2_ | p FDR=0.028 | ↓ |
| 132.0423 | 35.05 | Ethylmalonic acid | C_5_H_8_O_4_ | p FDR=0.016 | ↓ |
| 133.0375 | 26.69 | Aspartic acid | C_4_H_7_NO_4_ | p FDR=0.016 | ↓ |
| 146.0691 | 28.96 | Glutamine | C_5_H_10_N_2_O_3_ | p FDR=0.015 | ↓ |
| 146.1055 | 30.76 | Lysine | C_6_H_14_N_2_O_2_ | p FDR=0.022 | ↓ |
| 147.0532 | 29.96 | Glutamic acid | C_5_H_9_NO_4_ | SR=1.2 | ↓ |
| 148.0372 | 48.24 | Hydroxyglutaric acid | C_5_H_8_O_5_ | p FDR=0.026 | ↓ |
| 148.0736 | 27.69 | Mevalonic acid | C_6_H_12_O_4_ | p FDR=0.026 | ↑ |
| 150.0528 | 27.01 | Pentose | C_5_H_10_O_5_ | p FDR=0.034 | ↓ |
| 165.0790 | 29.79 | Phenylalanine | C_9_H_11_NO_2_ | p FDR=0.026 | ↓ |
| 180.0535 | 46.41 | Nicotinuric acid | C_8_H_8_N_2_O_3_ | p FDR=0.034 | ↓ |
| 192.0270 | 56.06 | Citric acid | C_6_H_8_O_7_ | SR=5.2;  VIP= 5.5; p FDR=0.002 | ↓ |
| 192.0270 | 31.70 | Acetylcholine | C_6_H_8_O_7_ | p FDR=0.034 | ↓ |
| 196.0583 | 52.09 | Gluconic acid | C_6_H_12_O_7_ | p FDR=0.024 | ↓ |
| 204.0899 | 25.66 | Tryptophan | C_11_H_12_N_2_O_2_ | p FDR=0.048 | ↓ |
| 210.0376 | 56.26 | Glucaric acid | C_6_H_10_O_8_ | SR=1.2 | ↓ |
| 216.1222 | 48.84 | Acetylarginine | C_8_H_16_N_4_O_3_ | p FDR=0.026 | ↓ |
| 220.0848 | 51.57 | Hydroxytryptophan | C_11_H_12_N_2_O_3_ | p FDR=0.012 | ↓ |
| 227.0906 | 32.29 | Deoxycytidine | C_9_H_13_N_3_O_4_ | p FDR=0.023 | ↓ |
| 267.0968 | 31.01 | Deoxyguanosine | C_10_H_13_N_5_O_4_ | SR=2.9; VIP=2.1 | ↓ |
| 267.0968 | 61.55 | Adenosine | C_10_H_13_N_5_O_4_ | p FDR=0.026 | ↓ |
| 298.1151 | 41.59 | Methylguanosine | C_11_H_16_N_5_O_5_ | p FDR=0.016 | ↓ |
| 298.2872 | 39.95 | Nonadecanoic acid | C_19_H_38_O_2_ | p FDR=0.016 | ↓ |
| 299.2824 | 35.25 | Sphingosine | C_18_H_37_NO_2_ | p FDR=0.026 | ↓ |
| 346.2144 | 50.79 | Deoxycortisol | C_21_H_30_O_4_ | p FDR=0.034 | ↓ |

SR: Selectivity Ratio; FDR: False Discovery Rate; VIP: Variable Importance in Projection.

Table 8. Putatively annotated metabolites detected with the use of GC-EI-QqQ/MS technique (experiment 2 – ccRCC *vs* healthy controls).

| **Monoisotopic mass [Da]** | **RT**  **[min]** | **Metabolite** | **Formula** | **Statistical test value** | **Regulation (ccRCC *vs* healthy patients)** |
| --- | --- | --- | --- | --- | --- |
| 136.0371 | 15.80 | Threonic acid | C_4_H_8_O_5_ | VIP=2.5;  p FDR= 0.002 | ↑ |
| 150.0528 | 17.83 | Pentose | C_5_H_10_O_5_ | p FDR=0.0000009 | ↑ |
| 152.0684 | 18.48 | Arabitol | C_5_H_12_O_5_ | VIP=1.2 | ↑ |
| 179.0582 | 23.23 | Hippuric acid | C_9_H_9_NO_3_ | p FDR=0.001 | ↓ |
| 221.0899 | 23.28 | Acetylglucosamine | C_8_H_15_NO_6_ | p FDR=0.00005 | ↑ |
| 342.1162 | 30.73 | Galactinol | C_12_H_22_O_11_ | p FDR=0.005 | ↑ |

SR: Selectivity Ratio; FDR: False Discovery Rate; VIP: Variable Importance in Projection.

Table 9. Putatively annotated metabolites detected with the use of CE-ESI(+)-TOF/MS technique (experiment 1 – RCC *vs* healthy controls).

| **Monoisotopic mass [Da]** | **MT**  **[min]** | **Metabolite** | **Mass error [ppm]** | **Formula** | **Formula match**  **factor** | **Statistical test value** | **Regulation**  **(RCC *vs* healthy patients)** |
| --- | --- | --- | --- | --- | --- | --- | --- |
| 75.0325 | 12.97 | Glycine | 6 | C_2_H_5_NO_2_ | 96% | VIP=2.4;  p FDR=0.008 | ↓ |
| 75.0690 | 10.54 | Trimethylamine oxide/ Aminopropanol | 7 | C_3_H_9_NO | 96% | VIP=3.1 | ↓ |
| 89.0479 | 13.90 | Alanine/Sarcosine | 2 | C_3_H_7_NO_2_ | 93% | p FDR=0.006 | ↓ |
| 113.0585 | 11.38 | Creatinine | 3 | C_4_H_7_N_3_O | 99% | p FDR=0.021 | ↑ |
| 117.0541 | 12.87 | Guanidinoacetic acid | 2 | C_3_H_7_N_3_O_2_ | 80% | VIP=1.3;  p FDR=0.009 | ↓ |
| 131.0704 | 13.68 | Creatine | 7 | C_4_H_9_N_3_O_2_ | 94% | VIP= 5.5;  p FDR= 0.014 | ↓ |
| 145.0736 | 19.63 | Aminooxohexanoic acid/ Acetamidobutanoic acid | 2 | C_6_H_11_NO_3_ | 77% | VIP=2.2;  p FDR=0.010 | ↓ |
| 155.0697 | 11.69 | Histidine | 0 | C_6_H_9_N_3_O_2_ | 99% | VIP=1.3 | ↓ |
| 159.1257 | 18.04 | Aminooctanoic acid | 1 | C_8_H_17_NO_2_ | 70% | p FDR=0.036 | ↓ |
| 169.0859 | 11.94 | Methylhistidine | 4 | C_7_H_11_N_3_O_2_ | 99% | VIP=1.7 | ↓ |
| 172.0477 | 15.27 | Hydantoinpropionic acid | 4 | C_6_H_8_N_2_O_4_ | 90% | p FDR=0.014 | ↓ |
| 174.0164 | 19.88 | Aconitic acid | 3 | C_6_H_6_O_6_ | 90% | p FDR=0.006 | ↑ |
| 174.0643 | 13.45 | Formiminoglutamic acid/Formylisoglutamine | 1 | C_6_H_10_N_2_O_4_ | 84% | p FDR= 0.006 | ↑ |
| 175.0953 | 14.11 | Argininic acid | 2 | C_6_H_13_N_3_O_3_ | 94% | p FDR=0.010 | ↓ |
| 202.1425 | 12.42 | Dimethylarginine (symetric/asymetric) | 2 | C_8_H_18_N_4_O_2_ | 99% | p FDR=0.015 | ↑ |
| 217.1310 | 14.75 | Propionylcarntine | 1 | C_10_H_19_NO_4_ | 80% | p FDR=0.007 | ↓ |
| 228.0746 | 15.10 | Deoxyuridine | 1 | C_9_H_12_N_2_O_5_ | 74% | p FDR=0.010 | ↑ |
| 237.0844 | 18.33 | Biopterin | 7 | C_9_H_11_N_5_O_3_ | 88% | p FDR=0.003 | ↑ |
| 243.0855 | 14.53 | Indolebutyric acid / Cytidine | 0 | C_9_H_13_N_3_O_5_ | 90% | p FDR=0.009 | ↑ |
| 246.0854 | 16.91 | Dihydrouridine | 0 | C_9_H_14_N_2_O_6_ | 82% | p FDR=0.006 | ↑ |
| 287.1117 | 13.97 | Ribosylhistidine | 0 | C_11_H_17_N_3_O_6_ | 82% | p FDR=0.038 | ↑ |
| 311.1228 | 17.73 | Dimethylguanosine | 0 | C_12_H_17_N_5_O_5_ | 94% | p FDR=0.010 | ↑ |
| 324.1555 | 13.59 | Galactosylhydroxylysine | 6 | C_12_H_24_N_2_O_8_ | 96% | p FDR=0.014 | ↑ |

SR: Selectivity Ratio; FDR: False Discovery Rate; VIP: Variable Importance in Projection.


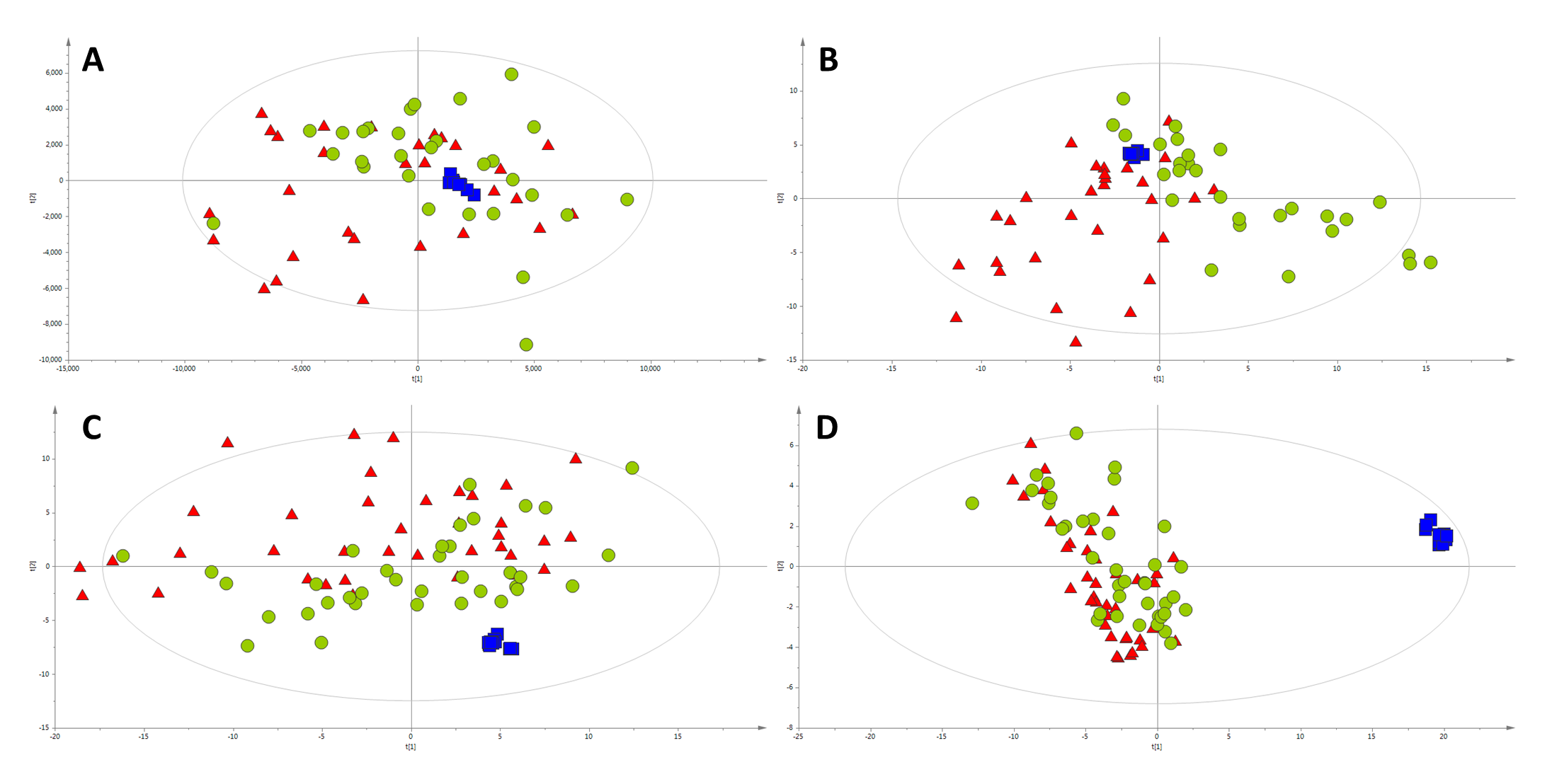


Figure 1.

PCA models built for data from analyses with the use of HPLC-TOF/MS technique. A – first experiment, ESI+ (R^2^=0.614); B – first experiment, ESI- (R^2^=0.300); C – second experiment, ESI+ (R^2^=0.554); D – second experiment, ESI- (R^2^=0.642). Blue boxes, green circles and red triangles represents QCs, healthy controls and cancer patients, respectively.


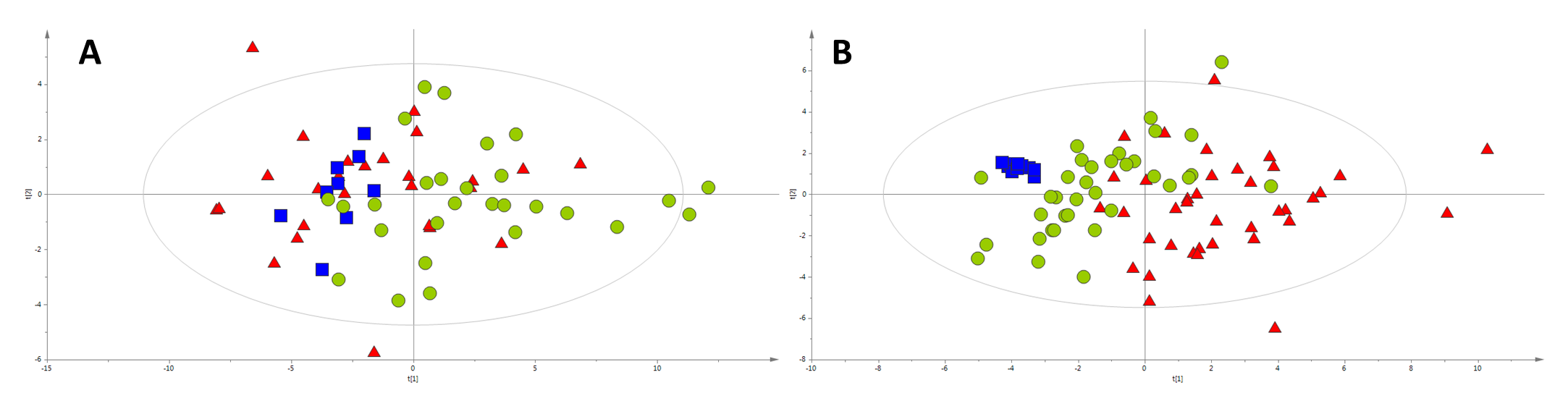


Figure 2.

PCA models built for data from analyses with the use of GC-QqQ/MS technique. A – first experiment (R^2^=0.315), B – second experiment (R^2^=0.231). Blue boxes, green circles and red triangles represents QCs, healthy controls and cancer patients, respectively.


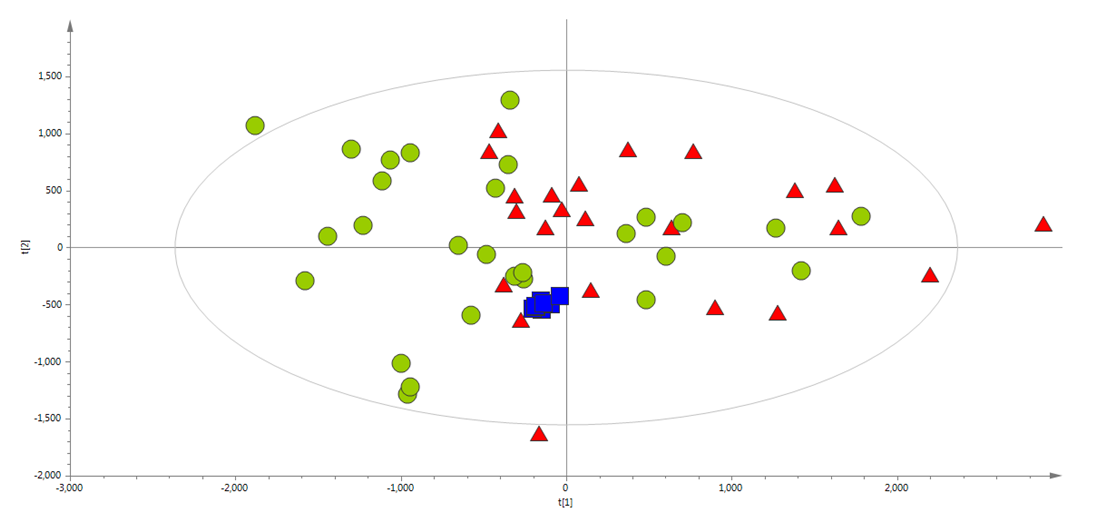


Figure 3.

PCA models built for data from analyses with the use of CE-ESI(+)-TOF/MS technique (R^2^=0.827). Blue boxes, green circles and red triangles represents QCs, healthy controls and cancer patients, respectively
